# Association of mucin family members with prognostic significance in pancreatic cancer patients: A meta-analysis

**DOI:** 10.1371/journal.pone.0269612

**Published:** 2022-06-16

**Authors:** Wei Xu, Man Zhang, Lu Liu, Minyue Yin, Chunfang Xu, Zhen Weng

**Affiliations:** 1 Department of Gastroenterology, The First Affiliated Hospital of Soochow University, Suzhou, Jiangsu, China; 2 Department of Emergency Medicine, The Affiliated Hospital of Xuzhou Medical University, Xuzhou, Jiangsu, China; 3 The First Affiliated Hospital of Soochow University, Suzhou, Jiangsu Province, China; 4 Cyrus Tang Hematology Center and Ministry of Education Engineering Center of Hematological Disease, The Collaborative Innovation Center of Hematology, Soochow University, Suzhou, China; OUHSC: The University of Oklahoma Health Sciences Center, UNITED STATES

## Abstract

**Objective:**

The role of biomarkers in the early diagnosis and prognosis prediction of tumors has been paid more and more attention by researchers. Mucins are markers that have been found to have an abnormal expression in many tumors in recent years, which have been proved to have a predictive effect on the prognosis of tumors such as cholangiocarcinoma and colon cancer. However, whether it can predict the prognosis of pancreatic cancer remains unknown. The purpose of our study is to investigate whether the mucins and their subtypes are related to the prognosis of patients with pancreatic cancer.

**Methods:**

We systematically searched the Pubmed, Embase, and Cochrane Library for all eligible studies on the relationship between mucin and the prognosis of patients with pancreatic cancer up to November 2021. We used R 4.12 to calculate the combined risk ratio (HR) and 95% confidence interval (CI). For studies that did not provide HR values, we used scientific methods to calculate their values as accurately as possible. We used fixed effect model due to low heterogeneity. Subgroup analysis and sensitivity analysis were used to study heterogeneity. The funnel plot and Egger test were used to test whether the publication bias existed. The trim and filling method were used to evaluate the impact of publication bias on the results of the study.

**Results:**

A total of 18 studies were included in this meta-analysis, including 4 subtypes of mucin family members and 1643 patients. There was a slight heterogeneity between studies (I2 = 24.4%, P = 0.14). Meta-analysis showed that MUC4 (HR = 2.04, 95%CI 1.21;3.45), MUC16 (HR = 2.10, 95%CI 1.31;3.37), and whole mucin (HR = 1.32, 95%CI 1.07;1.63). The expression level was negatively correlated with the prognosis of pancreatic cancer patients, MUC1 (HR = 1.09, 95%CI 0.77;1.54), MUC5 (HR = 1.03, 95%CI 0.47;2.25) The expression level was not related to the prognosis of pancreatic cancer patients.

**Conclusion:**

The meta-analysis demonstrated that the overall expression level of mucin and the expression levels of MUC4 and MUC16 were important prognostic predictors for pancreatic cancer patients. MUC1 and MUC5 had no predictive value for the prognosis of pancreatic cancer patients. Future studies should validate these and other promising biomarkers.

**Trial registration:**

PROSPERO registration number is CRD42021291962. https://www.crd.york.ac.uk/prospero/display_record.php?ID=CRD42021291962.

## 1. Introduction

Pancreatic cancer (PC) is a malignant tumor of the alimentary system with concealed pathogenesis, a high degree of malignancy, rapid progression, and poor prognosis. According to the location of pancreatic cancer, it is mainly divided into two clinical types: pancreatic head carcinoma and carcinoma of the pancreatic body and tail. Most pancreatic cancers originate from the pancreatic ductal glandular epithelium and form pancreatic ductal adenocarcinoma (PDAC). A few are from other sources such as mucinous cystadenocarcinoma, acinar cell carcinoma, and so on. Smoking, obesity, diabetes, and alcohol intake are recognized as the most important risk factors for pancreatic cancer [1]. In addition, many studies have also found that ABO blood group, infection, chronic pancreatitis, dietary habits, and so on may play a role in the pathogenesis and progression of pancreatic cancer [2]. According to statistics, pancreatic cancer is the third leading cause of cancer-related deaths in the United States with a 5-year survival rate of 10% [3]. It is expected to surpass breast cancer as the third leading cause of cancer death [4]. Surgery is the only hope for curing pancreatic cancer [5]. However, pancreatic cancer usually has no obvious symptoms in the early stage. Once patients have clinical symptoms such as abdominal or middle back pain, obstructive jaundice and weight loss [6], it means that most of them have entered the advanced stage, and it is often difficult for patients to have the opportunity to operate. Add insult to injury, pancreatic cancer is not sensitive to chemotherapy and radiotherapy. Even immunotherapy, which has made great progress in recent years, is still not effective in the treatment of pancreatic cancer. Looking for the factors that affect the prognosis of pancreatic cancer can predict the survival time of patients with pancreatic cancer and provide a new direction for basic research and clinical treatment.

Mucins are glycoproteins synthesized by epithelial cells. Dozens of mucin families have been found, including MUC1, MUC2, MUC4, MUC5, MUC6, MUC16, MUC20, and so on [7]. These proteins are mainly characterized by high levels of O-linked oligosaccharides, divided into two types: secretory mucin and membrane-anchored mucin, according to their mode of action and distribution. Mucin plays a protective role in the body. Once secreted, it forms a barrier that protects fragile epithelial cells from the extracellular environment and selects substances for epithelial cells to bind and ingest [8]. The investigators deem that there is a significant correlation between the expression level of the mucin family and various malignant tumors. In previous studies, it has been proved that the mucin family can be used as a diagnostic factor for various cancers [9]. Some members of the mucin family (such as MUC4) can predict the adverse clinical outcome of a variety of cancers (such as lung cancer, cholangiocarcinoma, colorectal cancer, etc.) [10–13]. However, the relationship between mucin family members and the prognosis of pancreatic cancer has not been determined. The purpose of this meta-analysis was to reveal the ability of mucin family members to predict the prognosis of pancreatic cancer and to provide strong evidence for the relationship between mucin family members and the prognosis of pancreatic cancer.

## 2. Methods

### 2.1 Search strategy and eligibility

Using the words “MUC,” “Mucin,” “pancreatic cancer,” and “prognosis” as keywords. A comprehensive and detailed search of PubMed, Embase, Cochrane and other medical databases was conducted to collect all the literature about the relationship between the members of the mucin family and the prognosis of pancreatic cancer. The deadline was November 2021. In order to make this study more reliable and rigorous, we set the inclusion and exclusion criteria of the literature according to the research needs. The inclusion criteria were as followed: (1) Explain the detection method of mucin expression level clearly, and this method could reflect the mucin expression level of pancreatic cancer correctly. (2) The diagnosis of pancreatic cancer was confirmed by imaging and pathological examination. (3) The study’s groups were divided into high expression groups and low expression groups according to the expression level of mucin. (4) The study must provide at least one of the following three items: explicit HR and 95%CI, survival curve from which HR can be extracted, or explicit HR and P value. (5) The study had high literature quality to ensure reliability, and the Newcastle-OttawaScale score was not less than 6. (6) In order to ensure the comparability of the members of the mucin family and facilitate the subgroup analysis in the future, only when the number of studies on a member of the mucin family was not less than 3, the research on that member will be included in the final analysis—each final included study needed to meet the above six criteria simultaneously. In addition, when the study meets any of the following exclusion criteria, it will be excluded. The exclusion criteria were as followed:(1) Animal experiments, abstracts, reviews, case reports, and studies that have nothing to do with the contents of this study. (2) The study of mucin combined with other mucins rather than alone. (3) The study only provided the survival curve. According to the standard method, the P value of the risk ratio extracted from the survival curve was quite different from that in the original study. (4) The study of other pancreatic tumors without pancreatic cancer or combining pancreatic cancer with other pancreatic tumors. The only study that met all inclusion criteria and did not meet any of the exclusion criteria would be included in the meta-analysis.

### 2.2 Data extraction and literature evaluation

The primary extracted data of the included study were as follows: title, first author name, publication time, mucin member type, number of control group and case group, region, the detection method of Mucin expression level, HR value, and 95%CI, the source of the sample population. Wei Xu and Man Zhang extracted and summarized independently from the research finally included in this study and compared after the extraction to prevent errors in the extraction process. For the divergent study, whether or not to be included was decided through discussion or consultation with the third author. For the lack of data in the study, contact the original author as much as possible to supplement. If the author could not be reached, then according to the impact on the study to decide whether to exclude. The Newcastle-Ottawa Scale scoring standard was used to evaluate the quality of the included research.

### 2.3 Statistical analysis

All data were analyzed with the R 4.1.2 software meta-package (version 4.9). If the study only provided a survival curve, we referred to Jayne F Tierney’s method [14], used the Engauge Digitizer 4.1 software to extract the data of the survival curve, and used the risk ratio calculation spreadsheet provided in Jayne F Tierney’s study to analyze the extracted data, then finally got the extracted HR and 95%CI. If the study only provided HR and P values, we calculated the range of 95% CI referring to the method of Douglas G Altman et al. [15]. Taking the low-level expression of mucin as a reference, the pooled HR and 95% CI of high-level expression of mucin were calculated to compare the overall survival time (OS) of patients with high-level and low-level expression of mucin. If HR > 1, it was considered that the high expression of mucin family indicated that pancreatic cancer patients had a poor prognosis. If HR < 1, then the high expression of mucin family indicated that pancreatic cancer patients had a better prognosis. 95% CI containing 1 equaled P < 0.05. The heterogeneity of the included literature was tested, and I^2^ statistics were used to evaluate the heterogeneity among the studies. It is statistically significant as P < 0.1. When I ^2^< 50%, it was considered that there did not have significant heterogeneity among studies, and the fixed effect model could be used. When I ^2^> 50%, it was considered significant heterogeneity among studies, and the random effect model could be used. Subgroup analysis would be carried out to search for the possible sources of heterogeneity. Sensitivity analysis was performed by omitting studies included one by one to estimate the possible sources of heterogeneity. Funnel plot and Egger’s test were conducted to evaluate whether this meta-analysis had publication bias. If P < 0.05, it represented that there might exist publication bias. All the statistical tests in this study were bilateral.

## 3. Results

### 3.1 Literature retrieval results

**Fig 1:**
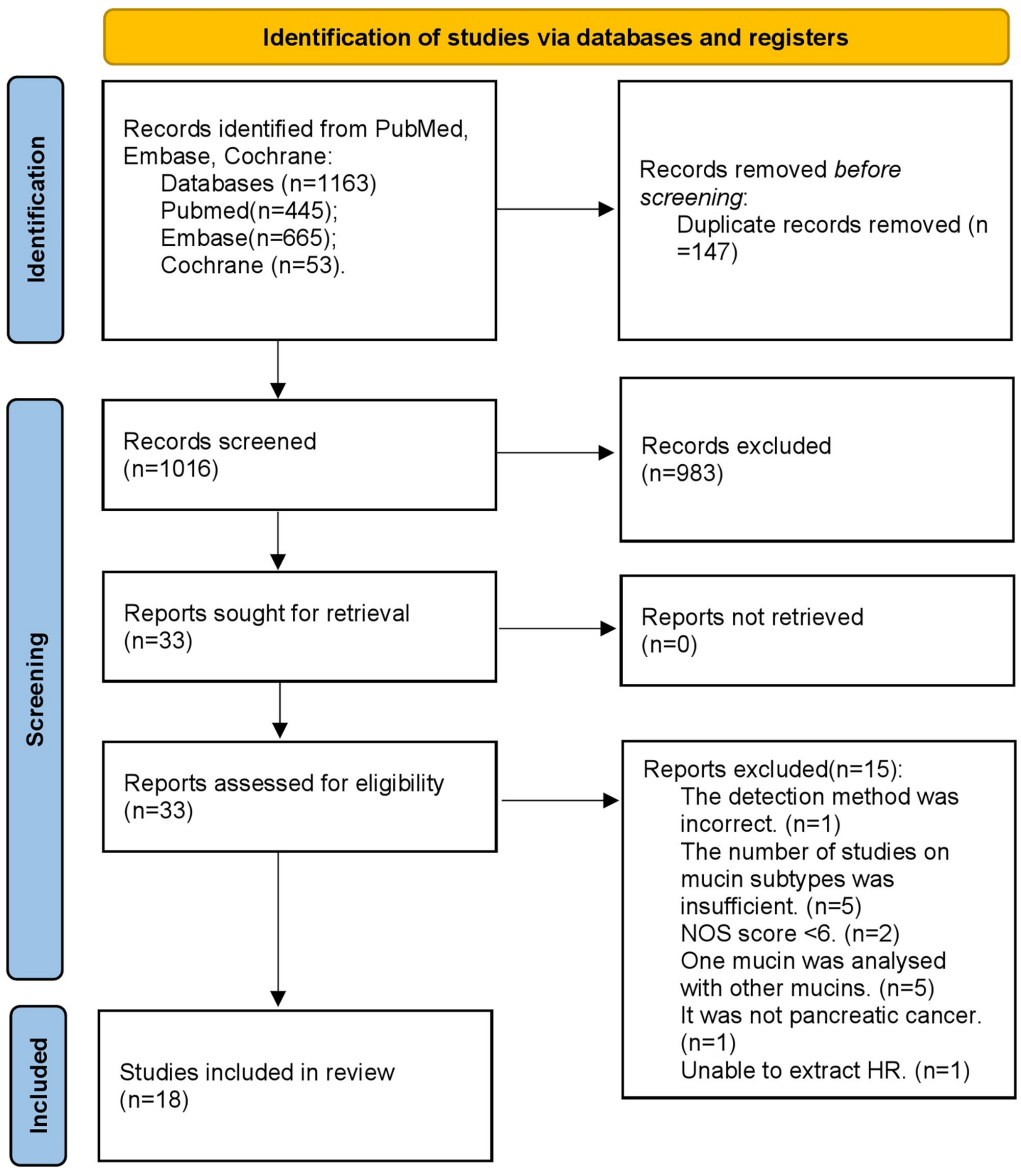
PRISMA flow chart for the identification of the included studies.

Through extensive review and strict compliance with the inclusion and exclusion criteria, a total of 18 studies [16–33] were included in this study (Fig 1), all of which were retrospective non-randomized studies. Except for the studies that did not specify the number of patients with high expression and low expression, 1643 patients were included in this study. Including 850 patients with high/positive expression of mucin and 793 patients with low/negative expression of mucin. Four subtypes of mucin family members were included in this study, including MUC1 (8 studies), MUC4 (6 studies), MUC5 (4 studies), and MUC16 (5 studies). A total of 464 patients were examined for MUC1 expression levels, including high expression/positive in 264 cases and low expression/negative in 200 cases. A total of 427 patients were examined for MUC4 expression levels, including high expression/positive in 160 cases and low expression/negative in 267 cases. A total of 228 patients were examined for MUC5 expression levels, including high expression/positive in 150 cases and low expression/negative in 78 cases. A total of 524 patients were examined for MUC16 expression levels, including high expression/positive in 276 cases and low expression/negative in 248 cases. The basic information extracted and the evaluation score of the research quality are summarized in Table 1.

**Table 1:** Characteristics of included studies.

study	year	MUC	high/positive	low/negative	HR^a^	95%CI	NOS	region	Method^b^	Location^c^	Data resource
Takikita, M.	2009	MUC1	139	15	1.6	0.9–3.0	8	USA	IHC	cytoplasm	SEER-TMA
		MUC5	99	55	1.4	1.0–2.1	8	USA	IHC	cytoplasm	SEER-TMA
Striefler, J. K.	2021	MUC1	23	115	2.17	1.35–3.45	7	Germany	IHC	cytoplasm	CONKO-001 Study
Sierzega, M.	2016	MUC1	NA*	NA	0.60	0.28–1.27	7	Poland	IHC	NA	clinical data
Jonckheere, N.	2020	MUC1	NA	NA	4.49	1.64–12.33	7	France	mRNA level	cytoplasm	TCGA
		MUC4	NA	NA	3.94	1.81–8.61	7	France	mRNA level	cytoplasm	TCGA
		MUC5B	NA	NA	4.38	1.76–10.9	7	France	mRNA level	cytoplasm	TCGA
		MUC16^#^	NA	NA	2.53	1.47–4.36	7	France	mRNA level	cytoplasm	TCGA
Hinoda, Y.^#^	2003	MUC1	39	31	1.82	1.11–2.98	7	Japan	IHC	membrane or cytoplasm	clinical data
Dotan, E.^#^	2016	MUC1	10	13	1.97	1.15–3.39	6	USA	IHC	cytoplasm	clinical data
Sato, K.^#^	2018	MUC1	14	15	2.47	1.25–4.87		Japan	mRNA	cytoplasm	clinical data
Yokoyama, S.	2016	MUC1	39	11	3.36	1.44–7.89	6	Japan	DNA methylation level	nucleus	clinical data
		MUC4	29	21	2.47	1.10–5.56	6	Japan	DNA methylation level	nucleus	clinical data
Zhu, Y.	2011	MUC4	29	28	2.50	1.33–4.70	6	China	mRNA level	cytoplasm	clinical data
Zhu, Y.	2014	MUC4	33	75	2.28	1.42–3.68	7	China	mRNA level	cytoplasm	clinical data
Yang^#^	2014	MUC4	48	29	1.75	1.10–2.78	6	China	IHC	NA	clinical data
Saitou, M.	2005	MUC4	21	114	1.96	1.13–3.38	6	Japan	IHC	Cytoplasm or cytomembrane	clinical data
Jinfeng, M.^#^	2003	MUC5AC	21	12	2.47	1.17–5.23	6	Japan	IHC	cytoplasm	clinical data
Yamasaki, H.^#^	2004	MUC5AC	30	11	0.53	0.2–1.41	6	Japan	IHC	Cytoplasm or cytomembrane	clinical data
Streppel, M. M.	2012	MUC16	119	81	2.00	1.36–2.94	7	USA	IHC	cytomembrane	clinical data
Shimizu, A.	2012	MUC16	41	62	1.94	1.13–3.31	7	Japan	IHC	membrane or cytoplasm	clinical data
Liang, C.	2017	MUC16	60	50	2.39	1.52–3.77	7	China	IHC	cytomembrane	clinical data
Fan, K.^#^	2018	MUC16c	56	55	1.98	1.14–3.45	8	China	IHC	NA	clinical data

* No information about the project was provided in the study.

^#^ The study did not provide HR or 95%CI. The HR and 95%CI were estimates.

^a^ The HR and 95%CI of overall survival (OS) were extracted. If both univariate and multivariate analysis HR existed, multivariate analysis HR was preferred.

^b^ It represented the detection method of mucin expression in the study. IHC referred to the immunohistochemical score to identify mucin expression and mRNA level referred to the expression level of mucin gene in the nucleus by checking the cytoplasmic mRNA content by qt-PCR.

^c^ It represented the localization of mucin.

### 3.2 Overall survival risk ratio

**Fig 2:**
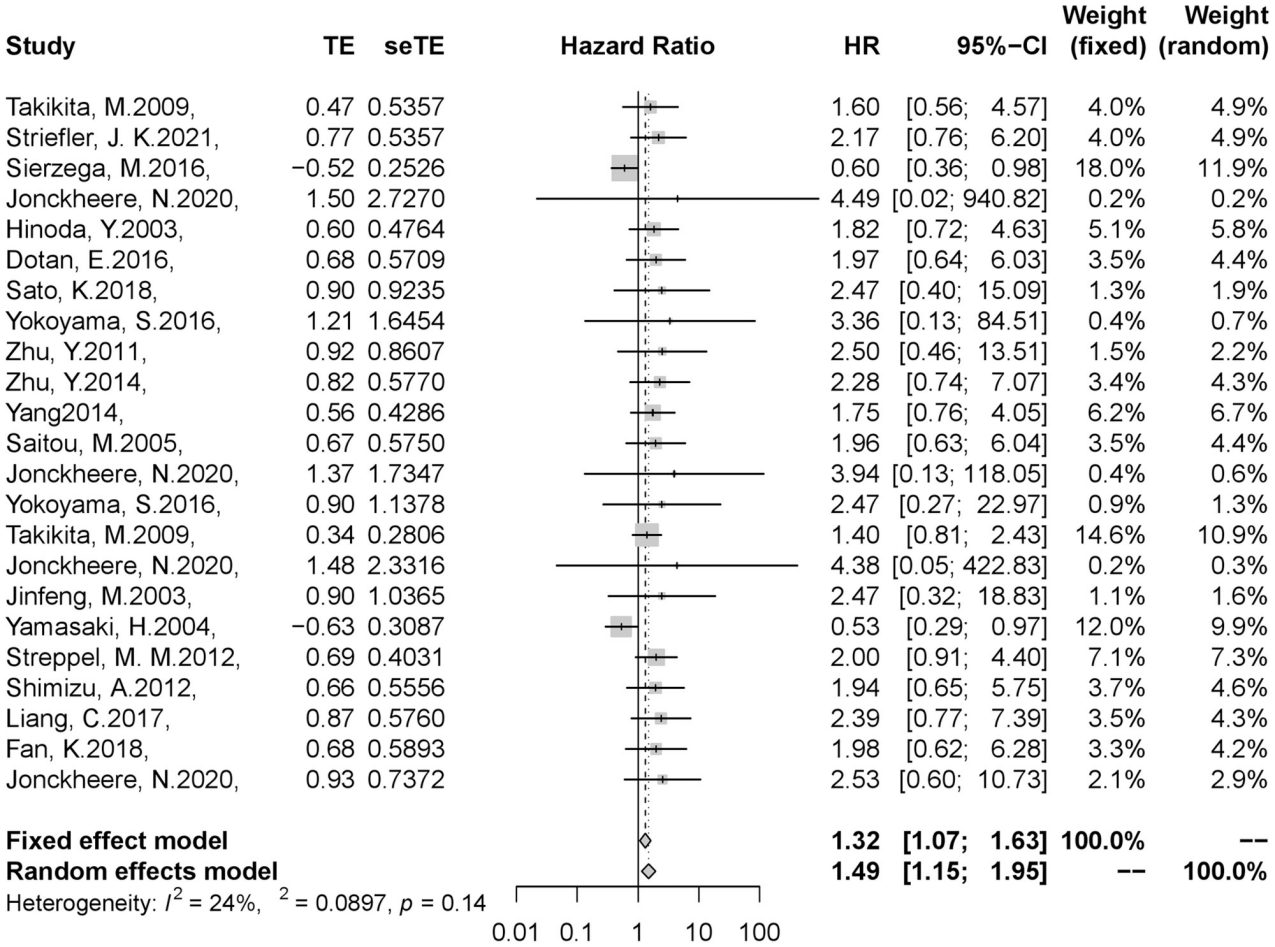
Results of the meta-analysis on pooled HR values. (A) Meta-analysis was performed by overall expression level of mucin. (B)Gray square represented HR of the study and horizontal lines represent 95%CI. Gray diamond represented the pooled HR. (C)We chose fixed effect model because heterogeneity was not significant in this meta-analysis (I^2^ = 24%, P = 0.14). Pooled HR = 1.32,95%CI 1.07–1.63.

The results of the meta-analysis showed that compared with the low expression/negative group, the pooled HR of the high expression/positive group was 1.32(95% CI, 1.07–1.63). The fixed effect model was used because of the slight heterogeneity in our study(I^2^ = 24.4%, P = 0.14). Therefore, the result indicated that the overall expression level of the mucin family was negatively correlated with the prognosis of patients with pancreatic cancer, which means that we can determine the prognosis and survival of patients by detecting the expression of mucin in clinical work (Fig 2).

### 3.3 Results of subgroup analysis and sensitivity analysis

**Fig 3:**
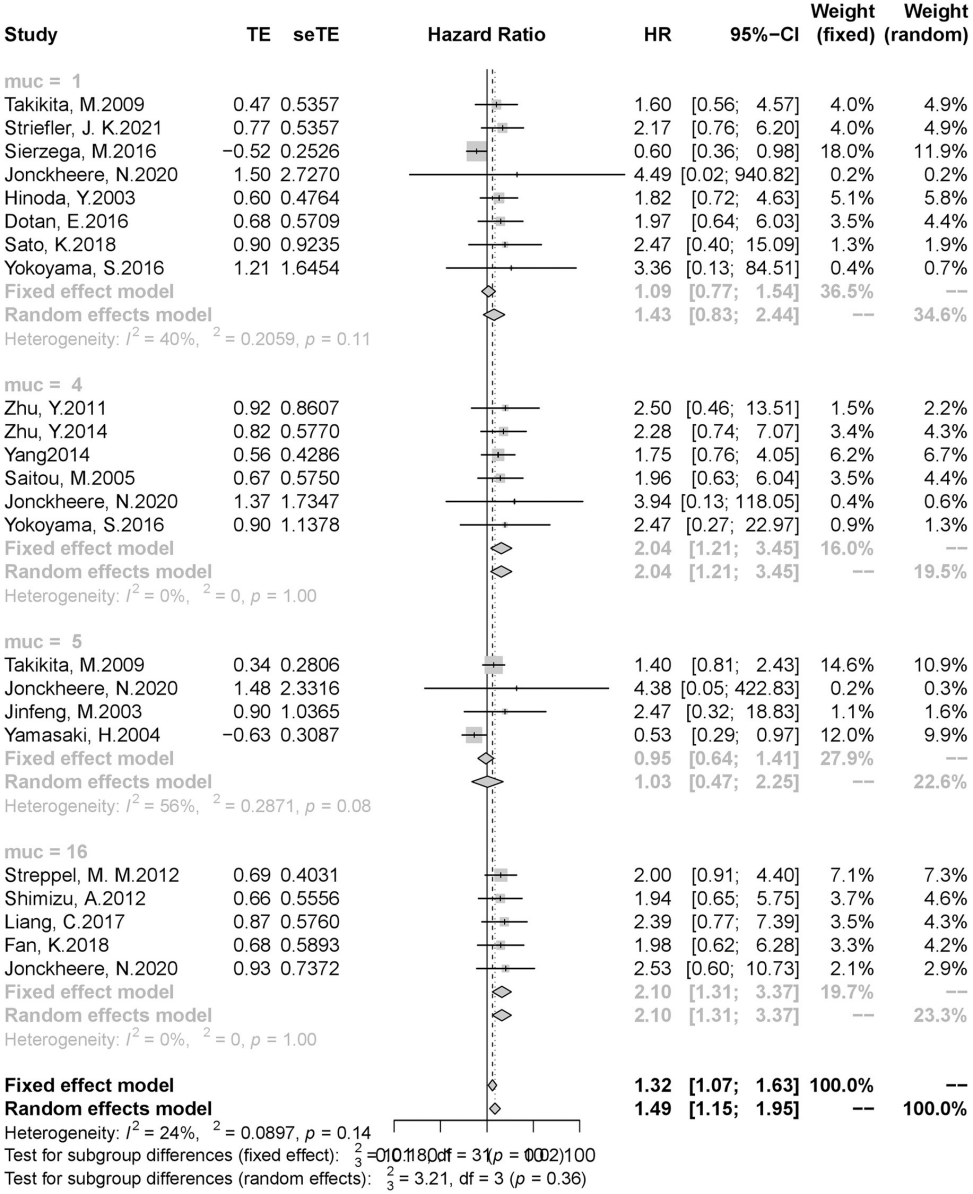
Result of subgroup analysis (1). (A)Subgroup analysis was performed by mucin subtype. (B)**muc = 1**, mucin1. **muc = 4,** mucin4. **muc = 5,** mucin5. **muc = 16**, mucin16. (C)MUC1: HR = 1.09,95% CI 0.77–1.54; MUC4: HR = 2.04,95%CI 1.21–3.45; MUC5: HR = 1.03,95%CI 0.47–2.25; MUC16: HR = 2.10,95%CI 1.31–3.37.

Although the heterogeneity of our study was not significant, in order to further explore the relationship between the expression level of each subtype of mucin family and the prognosis of patients with pancreatic cancer, we conducted subgroup analysis according to different subtypes of mucin. The results of subgroup analysis showed that the expression levels of MUC4(HR = 2.04,95%CI 1.21–3.45) and MUC16 (HR = 2.10,95%CI 1.31–3.37) were significantly correlated with the prognosis of patients with pancreatic cancer, suggesting that patients with high expression of mucin 4 and 16 often have a poor prognosis. There was no significant correlation between the expression of MUC1 (HR = 1.09,95% CI 0.77–1.54) and MUC5(HR = 1.03,95%CI 0.47–2.25) and the prognosis of patients with pancreatic cancer (Fig 3).

**Fig 4:**
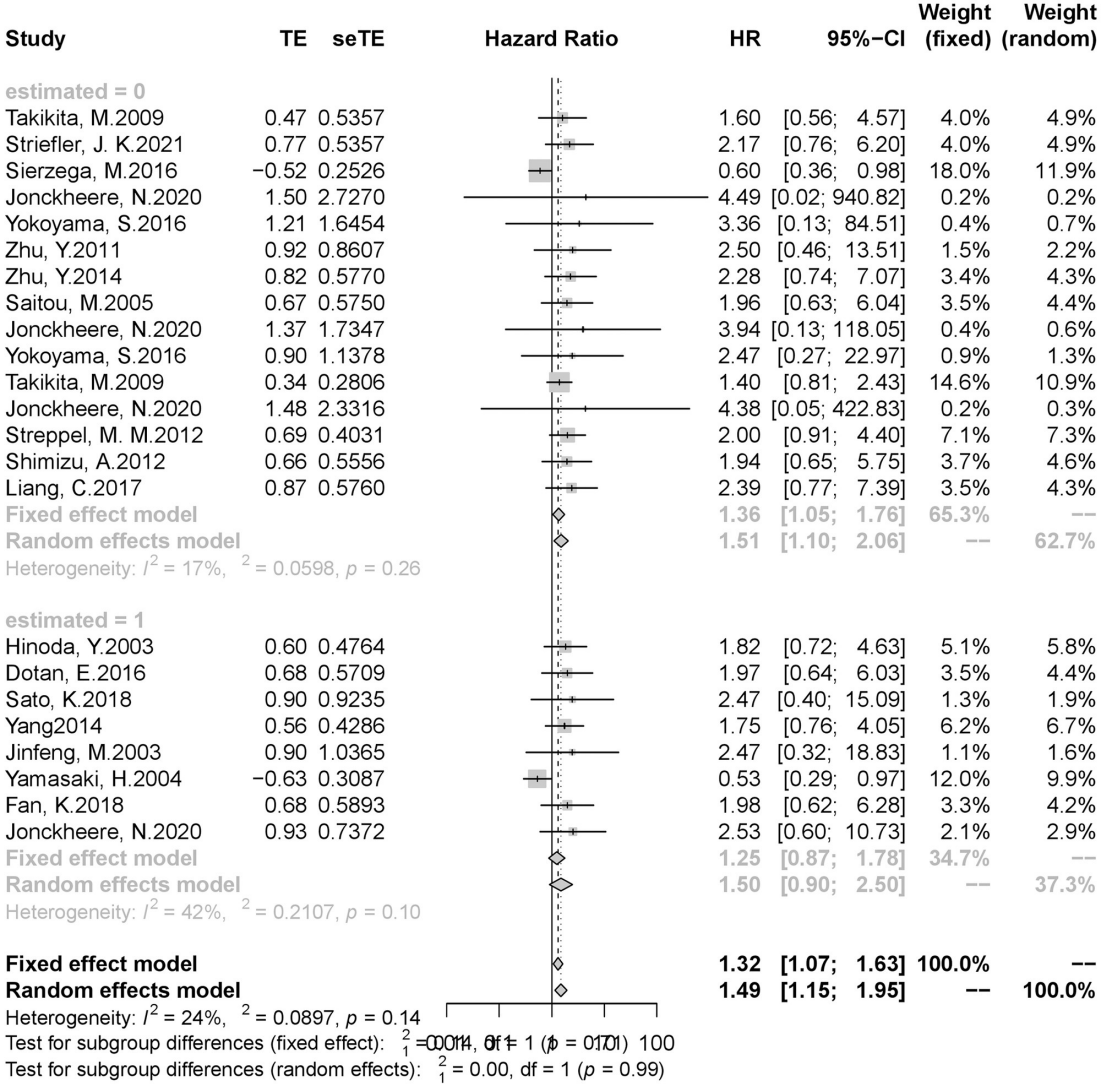
Result of subgroup analysis (2). (A)Subgroup analysis was performed based on whether HR and 95%CI was the estimated value. (B)**estimated = 0**, HR and 95%CI were provided directly. **estimated = 1**, HR or 95%CI were estimated. (C) **estimated = 0**: HR = 1.36,95%CI 1.05–1.76; **estimated = 1:** HR = 1.25,95%CI 0.87–1.78.

Since our study included several studies that did not provide HR and 95%CI directly, the inclusion of the effect values calculated by the estimated method in the study may reduce the study’s credibility. In order to observe the impact of the inclusion of estimates on this study, we conducted a subgroup analysis according to whether the effect value and 95%CI were provided directly. The result of subgroup analysis showed that pancreatic cancer patients with high/positive mucin expression still had a poor prognosis after removing the estimated study (HR = 1.36,95%CI 1.05–1.76). Therefore, it could be considered that the study of the estimated effect value we included did not significantly impact the results, and the original results were still credible (Fig 4).

**Fig 5:**
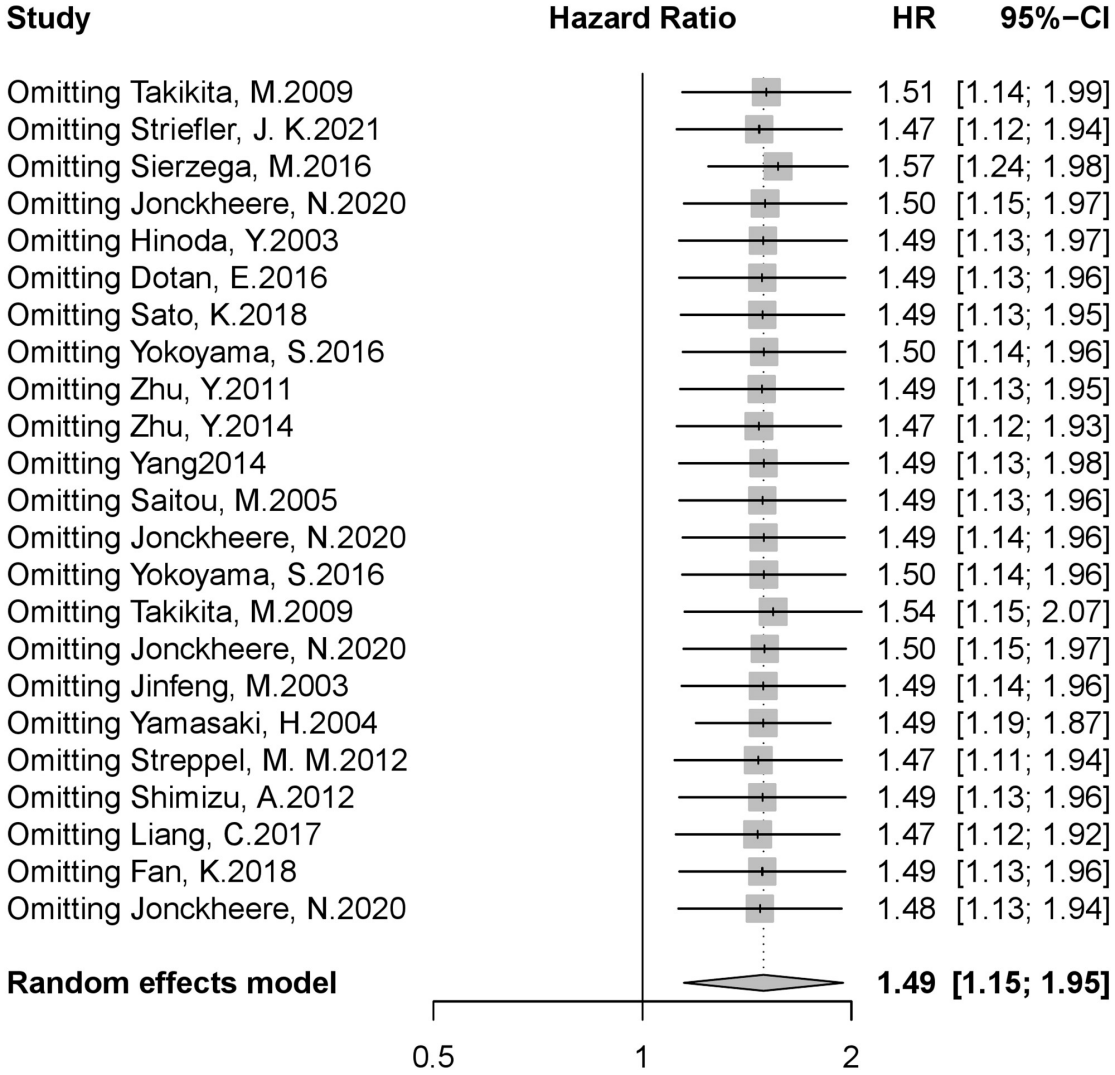
Result of sensitivity analysis. (A)The changes of pooled HR and 95%CI were observed by deleting the literature one by one. Sensitivity analysis showed the results was stable. (B) Pooled HR ranged from 1.47 to 1.57 and the 95% CI ranged from 1.11 to 2.07.

The changes of pooled HR and 95%CI were observed by deleting the literature one by one. If there was no significant change in HR and 95%CI after the deletion of a study (HR and 95%CI were still greater than 1), it indicates that the study has no significant effect on the study. A significant change in the overall results after the deletion of a study (HR and 95%CI are equal to or less than 1) showed that the study had a great impact on this study. The sensitivity analysis results suggested that excluding any study would not significantly change the pooled HR value, and 95% CI. Pooled HR ranged from 1.47 to 1.57, and the 95% CI was always greater than 1, suggesting that this study’s results were highly reliable (Fig 5).

### 3.4 Testing for publication bias

**Fig 6:**
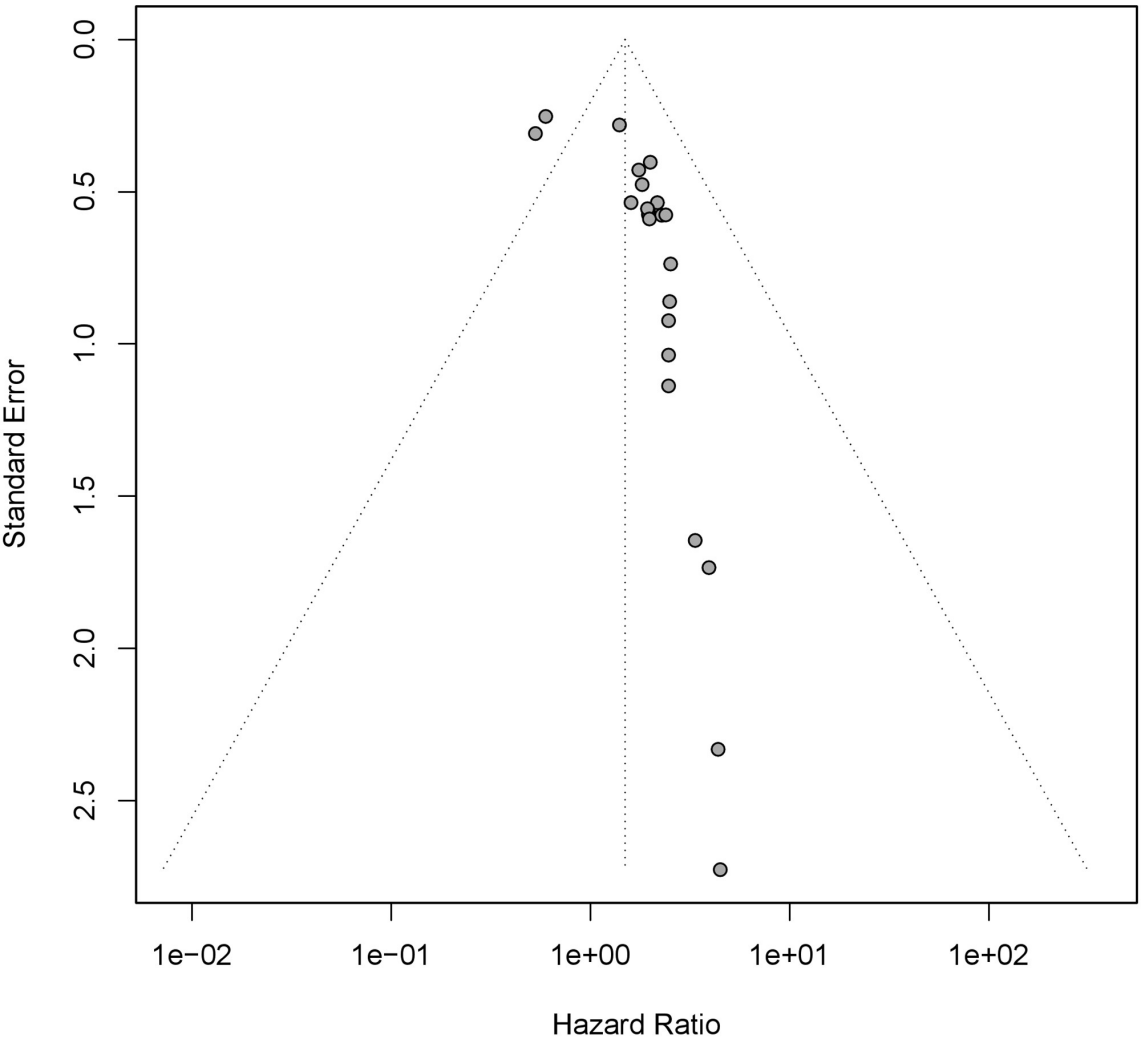
Funnel plot assess publication bias. The asymmetry on both sides of funnel plot suggested the existence of publication bias. Each point represents a separate study for the indicated association.

**Fig 7:**
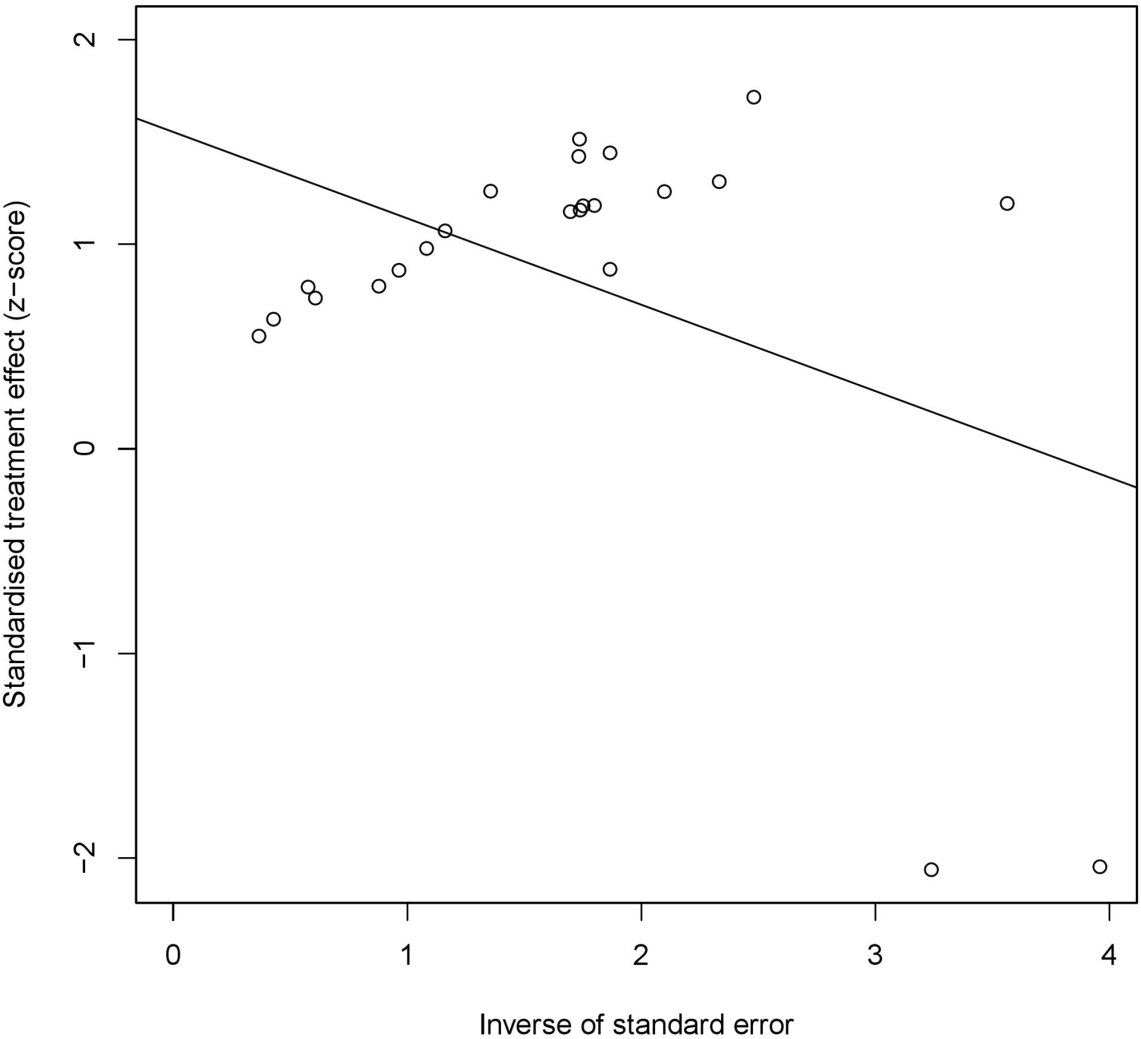
Result of Egger’s test. Egger’s test proved the possibility of publication bias (P < 0.05). Each point represents a separate study for the indicated association.

**Fig 8:**
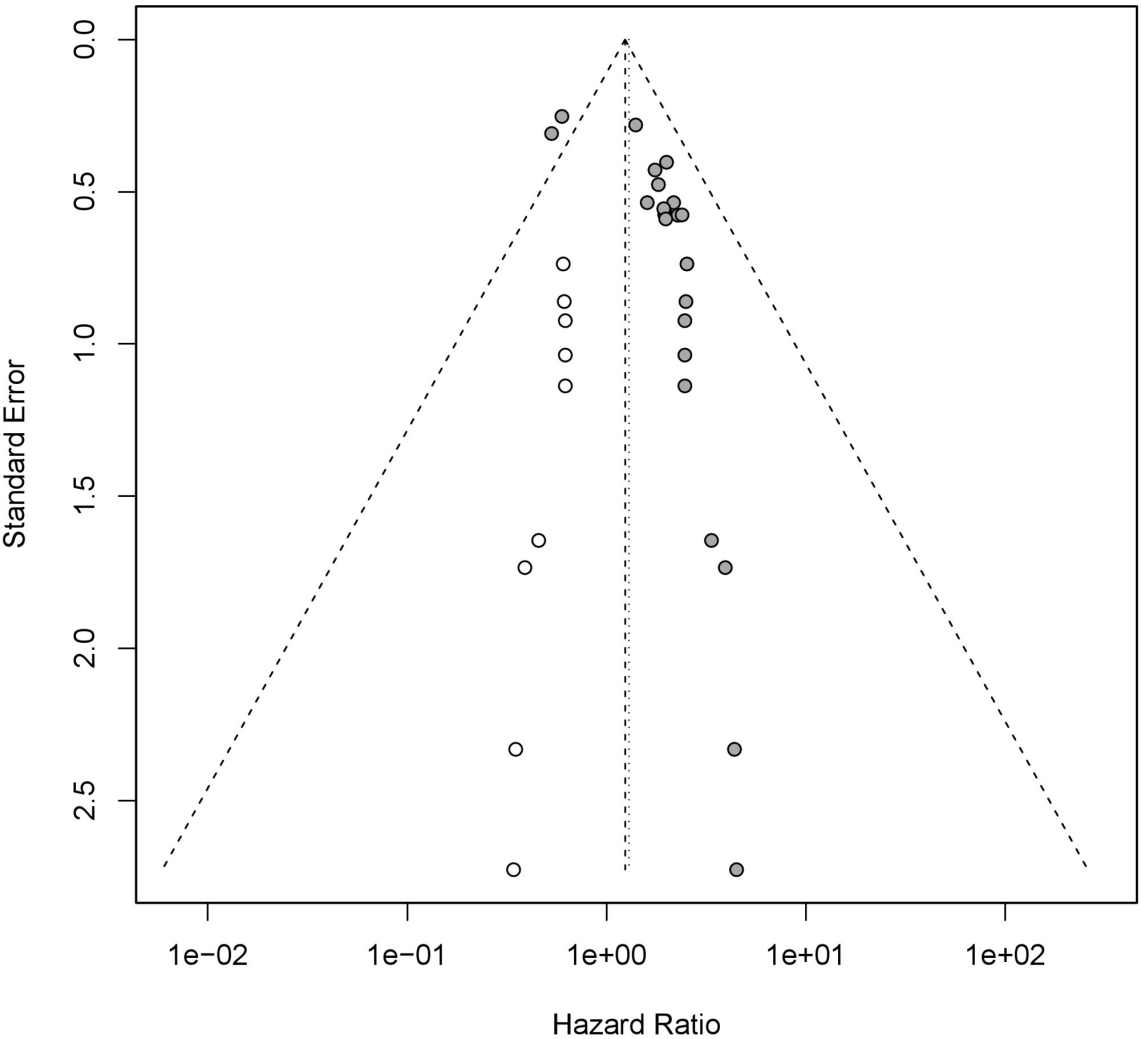
Publication bias was corrected by trim and filling method. (A)The hollow circle on the left side was supplementary studies by trim and filling method. (B)9 additional studies were supplemented to control publication bias, the result of which showed that the expression level of mucin family was still related to the prognosis of patients with pancreatic cancer (HR = 1.24,95%CI 1.01–1.52).

The funnel plot was used to detect the publication bias. We observed that the funnel plot was asymmetric (Fig 6), suggesting that publication bias might exist. Egger’s test (Fig 7) proved the possibility of publication bias (P < 0.05). Considering that the existence of publication bias might reduce the credibility of our study and lead to instability or even reversal of the results, we used trim and filling method to observe the effect of publication bias on this study (Fig 8). The result of trim and filling method supplemented 9 additional studies to control publication bias, showed that the expression level of mucin family was still related to the prognosis of patients with pancreatic cancer (HR = 1.24,95%CI 1.01–1.52), which further proved that the impact of publication bias on this study was not significant, and the results of our study had high credibility.

## 4. Discussion

Researchers have devoted themselves to studying the relationship between the expression level of mucin and human tumors since the discovery of mucin. In 2017, Ryan J. King et al. conducted a detailed and comprehensive study on the relationship between mucin and human tumors. In their studies, they found that various types of mucins increased expression in tumors through various forms (such as increased expression of mRNA, increased expression from scratch, increased copy number and reduced methylation), indicating that there has a link between mucin family and various types of cancer [34]. Mucin is usually divided into the transmembrane type and secretory type, and it or its fragments can be detected in blood examination after entering the blood. It can be used as a tumor marker to assist tumor diagnosis and predict the prognosis of tumor patients (such as CA125 [35,36], CA153 [37], CA199 [38], etc.). Many studies have shown that mucin plays an important role in diagnosing and prognosis of tumor diseases. However, there is no conclusion on the relationship between mucin and the prognosis of patients with pancreatic cancer. This study analyzed the relationship between the expression level of the mucin family and its subtypes (MUC1, MUC4, MUC5, MUC16) and the overall survival of patients with pancreatic cancer. The results showed that the overall expression level of mucin family and the patients with positive or high expression of MUC4 and MUC16 had a poor prognosis, indicating that mucin family can be used as a potential biomolecule to predict the prognosis of patients with pancreatic cancer.

The mechanism of the role of mucin in the occurrence and development of pancreatic cancer has not been fully elucidated. However, some of the mechanisms have been revealed after in-depth study. In normal cells, the promoter of the mucin gene is usually silenced because of methylation, and the mucin gene is also in a state of low expression. In the process of tumorigenesis, the promoter of the mucin gene will be demethylated under the action of methyltransferase DNMT1, which leads to the high expression of the mucin gene [39]. The high expression of the mucin gene leads to the overexpression of its product mucin, which is involved in the progression of pancreatic cancer through a series of pathways. Jonckheere, N. et al. considered MUC4 the ErbB2 ligand and TGF- β pathway target. The MUC4-ErbB2 complex can regulate the production of cell cycle inhibitor p27kip1 or cyclinD1 and play an essential role in the biological characteristics of pancreatic cancer cells and the progression of pancreatic cancer [40]. Researchers also found that Human epidermal growth factor receptor-2(HER2) may play an important role in the progression of pancreatic cancer promoted by MUC4. HER2 belongs to the ErbB family of receptor tyrosine kinases, which colocalizes with MUC4 in cell surface and cytoplasm. Silencing of MUC4 by transient or stable expression of MUC4-targeted short-interfering RNA led to the down-regulation of HER2 with a concomitant decrease in its phosphorylated form (pY1248-HER2). This reduces the ability of HER2 to regulate proliferation and metastasis by activating downstream mitogen-activated protein kinase (MAPK) and phosphoinositide-3-kinase/Akt pathways [41]. Bafna S. et al. have pointed out in their studies that MUC4 can exert its anti-apoptotic function through HER2/ extracellular signal to regulate kinase-dependent phosphorylation and inactivation of pro-apoptotic protein Bad, which makes pancreatic cancer cells resistant to gemcitabine, thus promoting cell survival [42]. Chen, S.H. et al. found that MUC16 is often over-expressed with MSLN in pancreatic cancer cells, which selectively induces the increase of matrix metalloproteinase (MMP)-7 through the p38MAPK-dependent pathway, which significantly enhances the motor and invasive ability of pancreatic cancer cells [43]. Fan, K et al. thought that the MUC16 terminal (MUC16c) activates the JAK2/STAT3 pathway through IL-6 secreted by the tumor, which promotes the expression of Foxp3 in tumor tissue and the accumulation of tumor-related Treg, thus promoting the invasion and immune escape of pancreatic cancer [17]. Moreover, Surendra K. Shukla et al. found that MUC16 knockdown pancreatic cancer cells had reduced glucose uptake and lactate secretion, reduced migration and invasion potential, and restored their original state after supplementation with lactate the final product of aerobic glycolysis. After further study, they concluded that this process was accomplished by inhibiting the PI3K-Akt-mTORC1 pathway [44]. These mechanisms provide further theoretical support for the relationship between the expression level of mucin and the prognosis of patients with pancreatic.

Paradoxically, our study did not seem to confirm this correlation though many studies suggested that MUC1 was associated with pancreatic cancer progression. MUC1 is a heterodimeric type I transmembrane protein that is normally expressed on the luminal surfaces of ductal epithelia. MUC1 is significantly overexpressed in tumor tissues. Although the receptor-like function of MUC1 provides mobility and environment-dependent adhesion / anti-adhesion function for cells, most of the carcinogenesis induced by MUC1 is due to its abnormal signal transduction interaction. In the case of tissue damage and loss of apical-basal polarity in tumor cells, MUC1 loses its apical localization and interacts with other receptor tyrosine kinases located on the base, thereby completely changing the signal transduction function of MUC1, thereby significantly regulating transcriptional regulation and other carcinogenic functions [45]. In Pankaj K. Singh’s study, they pointed out that the overexpression of MUC1 down-regulated the interaction between HGF-stimulated Met and conventional downstream signal transduction, thereby inhibiting movement and invasion. P53 and MMP1 play important roles in this process. Therefore, they scientifically proved that MUC1 was a regulator of Met signal transduction in pancreatic adenocarcinoma cells [46]. MUC1 was also implicated in pancreatic cancer glycolysis. MUC1 could promote the recruitment of HIF-1α and p300 on glycolysis gene promoter in the hypoxic environments and regulate multiple metabolites intermediates in glucose and amino acid metabolic pathways [47]. In addition to the study on the carcinogenic mechanism, researchers also found that the expression of MUC1 promoted the drug resistance of pancreatic cancer. MUC1 is involved in the vital process of HIF-1α regulating tumor glucose metabolism, leading to increased dependence of tumor cells on glucose and the increase of corresponding pyrimidines, thereby improving the intrinsic level of dCTP. The increase of dCTP level will reduce the effective level of gemcitabine through molecular competition [48]. In addition, MUC1 expression can also reduce radiation-induced cytotoxicity and DNA damage in pancreatic cancer cells by enhancing glycolysis, pentose phosphate pathway, and nucleotide biosynthesis, thereby making tumors insensitive to radiotherapy [49]. In fact, our findings did not contradict previous studies. Although we believed that MUC1 expression was not associated with the prognosis of pancreatic cancer patients, we did not deny the role of MUC1 as a potential carcinogenic factor in the development and drug resistance of pancreatic cancer. Similarly, we did not deny the multiple links between other mucins (MUC5) and pancreatic cancer.

In addition to being a biomarker, more and more studies have shown that mucins play an important role in tumorigenesis, immune escape, metastasis, and chemotherapy resistance. Therefore, they are the targets of developing new therapies and enhancing cytotoxic drugs [50]. As mentioned above, MUC1 can regulate glucose metabolism of pancreatic cancer through the HIF-1alpha pathway to induce resistance to gemcitabine, and targeted therapy for MUC1 may enhance the efficacy of gemcitabine for pancreatic cancer [48]. Researchers in the University of Nebraska Medical Center recently found that MUC5AC, as a link between β-catenin and c-Myc, increased glutamine uptake, which was used in pancreatic cancer cells. Co-targeting MUC1 with gemcitabine can improve the therapeutic effect of pancreatic cancer [51]. Therefore, mucin can be used as a biomarker to predict the prognosis of patients and as a therapeutic target to improve the efficacy of pancreatic cancer patients.

This study proved that the expression of mucin family and its subtypes were correlated with the prognosis of pancreatic cancer patients, and heterogeneity was not significant. There might exist publication bias, but we prove that it could have no significant effect on the results. Although we used estimated values to replace some included studies’ effect values, we thought it had no significant impact on the overall results after subgroup analysis, so the overall results were reliable. However, the shortcomings of our study could not be ignored. All the studies included were case-control studies, and the number of cases was small, so it was difficult to avoid selection bias and recall bias. Some of the studies used the expression effect of mucin fragments instead of the whole mucin expression effect, which might lead to the opposite outcome once the two have a different biological effect. Due to the lack of research data, other mucin subtypes such as MUC2, MUC6, and MUC7and so on could not be included in the meta-analysis. Most studies used the immunohistochemical score to evaluate the expression level of mucin, which was somewhat subjective. Some studies reflected the expression level of mucin by mRNA level and degree of promoter methylation, so the evaluation methods are quite different. In addition, the localization of mucin in pancreatic cancer cells varies from study to study. Some studies evaluated the expression level of mucin on the cell membrane, while others evaluated it in the cytoplasm and nucleus. Therefore, the different localization of mucin in pancreatic cancer cells might also lead to inaccurate results.

Our study is consistent with the previous study on the relationship between mucin and the prognosis of pancreatic cancer, affirming the study on the relationship between the two and supporting further research to explore the predictive ability of mucin of various subtypes to the prognosis of patients with pancreatic cancer. In addition, future research should focus on making mucin detection simpler and making mucin detection more widely used in predicting the prognosis of pancreatic cancer patients. More studies are needed to further clarify the mechanism of mucin in the progression of pancreatic cancer. In addition to being a biomarker to predict the prognosis of patients with pancreatic cancer, mucin may become a new target in treating pancreatic cancer in the future.

## Supporting information

S1 Checklist PRISMA 2020 checklist. (DOCX)

## References

[1] Klein AP. Pancreatic cancer epidemiology: understanding the role of lifestyle and inherited risk factors. Nature reviews Gastroenterology & hepatology. 2021;18(7):493–502. Epub 2021/05/19. doi: 10.1038/s41575-021-00457-x . 34002083 PMC9265847

[2] Cai J, Chen H, Lu M, Zhang Y, Lu B, You L, et al. Advances in the epidemiology of pancreatic cancer: Trends, risk factors, screening, and prognosis. Cancer Lett. 2021;520:1–11. Epub 2021/07/04. doi: 10.1016/j.canlet.2021.06.027 . 34216688

[3] Park W, Chawla A, O’Reilly EM. Pancreatic Cancer: A Review. Jama. 2021;326(9):851–62. Epub 2021/09/22. doi: 10.1001/jama.2021.13027 . 34547082 PMC9363152

[4] Bray F, Ferlay J, Soerjomataram I, Siegel RL, Torre LA, Jemal A. Global cancer statistics 2018: GLOBOCAN estimates of incidence and mortality worldwide for 36 cancers in 185 countries. CA Cancer J Clin. 2018;68(6):394–424. Epub 2018/09/13. doi: 10.3322/caac.21492 . 30207593

[5] Wray CJ, Ahmad SA, Matthews JB, Lowy AM. Surgery for pancreatic cancer: recent controversies and current practice. Gastroenterology. 2005;128(6):1626–41. Epub 2005/05/12. doi: 10.1053/j.gastro.2005.03.035 . 15887155

[6] Vincent A, Herman J, Schulick R, Hruban RH, Goggins M. Pancreatic cancer. Lancet (London, England). 2011;378(9791):607–20. Epub 2011/05/31. doi: 10.1016/S0140-6736(10)62307-0 . 21620466 PMC3062508

[7] Dekker J, Rossen JW, Büller HA, Einerhand AW. The MUC family: an obituary. Trends in biochemical sciences. 2002;27(3):126–31. Epub 2002/03/15. doi: 10.1016/s0968-0004(01)02052-7 . 11893509

[8] Strous GJ, Dekker J. Mucin-type glycoproteins. Critical reviews in biochemistry and molecular biology. 1992;27(1–2):57–92. Epub 1992/01/01. doi: 10.3109/10409239209082559 . 1727693

[9] Wang S, You L, Dai M, Zhao Y. Quantitative assessment of the diagnostic role of mucin family members in pancreatic cancer: a meta-analysis. Annals of Translational Medicine. 2021;9(3). doi: 10.21037/atm-20-5606 33708819 PMC7940915

[10] Huang X, Wang X, Lu SM, Chen C, Wang J, Zheng YY, et al. Clinicopathological and prognostic significance of MUC4 expression in cancers: Evidence from meta-analysis. International Journal of Clinical and Experimental Medicine. 2015;8(7):10274–83. 26379819 PMC4565202

[11] Li B, Tang H, Zhang A, Dong J. Prognostic Role of Mucin Antigen MUC4 for Cholangiocarcinoma: A Meta-Analysis. PLoS One. 2016;11(6):e0157878. Epub 2016/06/16. doi: 10.1371/journal.pone.0157878 . 27305093 PMC4909222

[12] Niv Y, Ho SB, Fass R, Rokkas T. Mucin Expression in the Esophageal Malignant and Pre-malignant States: A Systematic Review and Meta-analysis. Journal of clinical gastroenterology. 2018;52(2):91–6. Epub 2017/07/12. doi: 10.1097/MCG.0000000000000863 . 28697153

[13] Niv Y, Rokkas T. Mucin Expression in Colorectal Cancer (CRC): Systematic Review and Meta-Analysis. Journal of clinical gastroenterology. 2019;53(6):434–40. Epub 2018/05/22. doi: 10.1097/MCG.0000000000001050 . 29782466

[14] Tierney JF, Stewart LA, Ghersi D, Burdett S, Sydes MR. Practical methods for incorporating summary time-to-event data into meta-analysis. Trials. 2007;8:16. Epub 2007/06/09. doi: 10.1186/1745-6215-8-16 . 17555582 PMC1920534

[15] Altman DG, Bland JM. How to obtain the confidence interval from a P value. BMJ (Clinical research ed). 2011;343:d2090. Epub 2011/08/10. doi: 10.1136/bmj.d2090 . 21824904

[16] Dotan E, Alpaugh RK, Ruth K, Negin BP, Denlinger CS, Hall MJ, et al. Prognostic Significance of MUC-1 in Circulating Tumor Cells in Patients with Metastatic Pancreatic Adenocarcinoma. Pancreas. 2016;45(8):1131–5. doi: 10.1097/MPA.0000000000000619 26967453 PMC4983223

[17] Fan K, Yang C, Fan Z, Huang Q, Zhang Y, Cheng H, et al. MUC16 C terminal-induced secretion of tumor-derived IL-6 contributes to tumor-associated Treg enrichment in pancreatic cancer. Cancer Letters. 2018;418:167–75. doi: 10.1016/j.canlet.2018.01.017 29337110

[18] Hinoda Y, Ikematsu Y, Horinochi M, Sato S, Yamamoto K, Nakano T, et al. Increased expression of MUC1 in advanced pancreatic cancer. Journal of gastroenterology. 2003;38(12):1162–6. doi: 10.1007/s00535-003-1224-6 14714254

[19] Jinfeng M, Kimura W, Hirai I, Sakurai F, Moriya T, Mizutani M. Expression of MUC5AC and MUC6 in invasive ductal carcinoma of the pancreas and relationship with prognosis. International journal of gastrointestinal cancer. 2003;34(1):9–18. doi: 10.1385/IJGC:34:1:09 15235131

[20] Jonckheere N, Auwercx J, Bachir EH, Coppin L, Boukrout N, Vincent A, et al. Unsupervised hierarchical clustering of pancreatic adenocarcinoma dataset from TCGA defines a mucin expression profile that impacts overall survival. Cancers. 2020;12(11):1–17. doi: 10.3390/cancers12113309 33182511 PMC7697168

[21] Liang C, Qin Y, Zhang B, Ji S, Shi S, Xu W, et al. Oncogenic KRAS targets MUC16/CA125 in pancreatic ductal adenocarcinoma. Molecular Cancer Research. 2017;15(2):201–12. doi: 10.1158/1541-7786.MCR-16-0296 28108627

[22] Saitou M, Goto M, Horinouchi M, Tamada S, Nagata K, Hamada T, et al. MUC4 expression is a novel prognostic factor in patients with invasive ductal carcinoma of the pancreas. Journal of clinical pathology. 2005;58(8):845–52. doi: 10.1136/jcp.2004.023572 16049287 PMC1770880

[23] Sato K, Mori R, Hiroshima Y, Oba MS, Matsuyama R, Bouvet M, et al. RT-PCR of peritoneal washings predicts peritoneal pancreatic cancer recurrence. Journal of Surgical Research. 2018;226:122–30. doi: 10.1016/j.jss.2017.11.009 29661277

[24] Shimizu A, Hirono S, Tani M, Kawai M, Okada K, Miyazawa M, et al. Coexpression of MUC16 and mesothelin is related to the invasion process in pancreatic ductal adenocarcinoma. Cancer Sci. 2012;103(4):739–46. Epub 2012/02/11. doi: 10.1111/j.1349-7006.2012.02214.x . 22320398 PMC7659350

[25] Sierzega M, Młynarski D, Tomaszewska R, Kulig J. Semiquantitative immunohistochemistry for mucin (MUC1, MUC2, MUC3, MUC4, MUC5AC, and MUC6) profiling of pancreatic ductal cell adenocarcinoma improves diagnostic and prognostic performance. Histopathology. 2016;69(4):582–91. doi: 10.1111/his.12994 27165582

[26] Streppel MM, Vincent A, Mukherjee R, Campbell NR, Chen SH, Konstantopoulos K, et al. Mucin 16 (cancer antigen 125) expression in human tissues and cell lines and correlation with clinical outcome in adenocarcinomas of the pancreas, esophagus, stomach, and colon. Hum Pathol. 2012;43(10):1755–63. Epub 2012/05/01. doi: 10.1016/j.humpath.2012.01.005 . 22542127 PMC3547617

[27] Striefler JK, Riess H, Lohneis P, Bischoff S, Kurreck A, Modest DP, et al. Mucin-1 Protein Is a Prognostic Marker for Pancreatic Ductal Adenocarcinoma: Results From the CONKO-001 Study. Frontiers in Oncology. 2021;11. doi: 10.3389/fonc.2021.670396 34386419 PMC8354141

[28] Takikita M, Altekruse S, Lynch CF, Goodman MT, Hernandez BY, Green M, et al. Associations between selected biomarkers and prognosis in a population-based pancreatic cancer tissue microarray. Cancer Res. 2009;69(7):2950–5. Epub 2009/03/12. doi: 10.1158/0008-5472.CAN-08-3879 . 19276352 PMC2711977

[29] Yamasaki H, Ikeda S, Okajima M, Miura Y, Asahara T, Kohno N, et al. Expression and localization of MUC1, MUC2, MUC5AC and small intestinal mucin antigen in pancreatic tumors. International journal of oncology. 2004;24(1):107–13. 14654947

[30] Yang K, He M, Cai Z, Ni C, Deng J, Ta N, et al. A decrease in miR-150 regulates the malignancy of pancreatic cancer by targeting c-Myb and MUC4. Pancreas. 2015;44(3):370–9. doi: 10.1097/MPA.0000000000000283 25522282

[31] Yokoyama S, Higashi M, Kitamoto S, Oeldorf M, Knippschild U, Kornmann M, et al. Aberrant methylation of MUC1 and MUC4 promoters are potential prognostic biomarkers for pancreatic ductal adenocarcinomas. Oncotarget. 2016;7(27):42553–65. doi: 10.18632/oncotarget.9924 27283771 PMC5173155

[32] Zhu Y, Zhang JJ, Xie KL, Tang J, Liang WB, Zhu R, et al. Specific-detection of clinical samples, systematic functional investigations, and transcriptome analysis reveals that splice variant MUC4/Y contributes to the malignant progression of pancreatic cancer by triggering malignancy-related positive feedback loops signaling. Journal of translational medicine. 2014;12:309. Epub 2014/11/05. doi: 10.1186/s12967-014-0309-8 . 25367394 PMC4236435

[33] Zhu Y, Zhang JJ, Zhu R, Zhu Y, Liang WB, Gao WT, et al. The increase in the expression and hypomethylation of MUC4 gene with the progression of pancreatic ductal adenocarcinoma. Medical Oncology. 2011;28(SUPPL. 1):S175–S84. doi: 10.1007/s12032-010-9683-0 20922503

[34] King RJ, Yu F, Singh PK. Genomic alterations in mucins across cancers. Oncotarget. 2017;8(40):67152–68. doi: 10.18632/oncotarget.17934 . 28978023 PMC5620163

[35] Liu L, Xu HX, Wang WQ, Wu CT, Xiang JF, Liu C, et al. Serum CA125 is a novel predictive marker for pancreatic cancer metastasis and correlates with the metastasis-associated burden. Oncotarget. 2016;7(5):5943–56. Epub 2016/01/09. doi: 10.18632/oncotarget.6819 . 26745601 PMC4868732

[36] Liu L, Xu H, Wang W, Wu C, Chen Y, Yang J, et al. A preoperative serum signature of CEA+/CA125+/CA19-9 ≥ 1000 U/mL indicates poor outcome to pancreatectomy for pancreatic cancer. International journal of cancer. 2015;136(9):2216–27. Epub 2014/10/03. doi: 10.1002/ijc.29242 . 25273947

[37] Li X, Xu Y, Zhang L. Serum CA153 as biomarker for cancer and noncancer diseases. Progress in molecular biology and translational science. 2019;162:265–76. Epub 2019/03/25. doi: 10.1016/bs.pmbts.2019.01.005 . 30905456

[38] Akagi J, Takai E, Tamori Y, Nakagawa K, Ogawa M. CA19-9 epitope a possible marker for MUC-1/Y protein. Int J Oncol. 2001;18(5):1085–91. Epub 2001/04/11. doi: 10.3892/ijo.18.5.1085 . 11295060

[39] Siedow A, Szyf M, Gratchev A, Kobalz U, Hanski ML, Bumke-Vogt C, et al. De novo expression of the Muc2 gene in pancreas carcinoma cells is triggered by promoter demethylation. Tumor Biology. 2002;23(1):54–60. doi: 10.1159/000048689 11893907

[40] Jonckheere N, Skrypek N, Saint-Laurent N, Dumont P, Susini C, Van Seuningen I. Knocking down of the MUC4 membranebound mucin and its membrane partner ErbB2 in human pancreatic cancer cells alter their in vitro and in vivo tumor properties. Pancreatology: official journal of the International Association of Pancreatology (IAP) [et al]. 2010;10(2–3):336.

[41] Chaturvedi P, Singh AP, Chakraborty S, Chauhan SC, Bafna S, Meza JL, et al. MUC4 mucin interacts with and stabilizes the HER2 oncoprotein in human pancreatic cancer cells. Cancer research. 2008;68(7):2065–70. doi: 10.1158/0008-5472.CAN-07-6041 . 18381409 PMC2835497

[42] Bafna S, Kaur S, Momi N, Batra SK. Pancreatic cancer cells resistance to gemcitabine: the role of MUC4 mucin. Br J Cancer. 2009;101(7):1155–61. Epub 2009/09/10. doi: 10.1038/sj.bjc.6605285 . 19738614 PMC2768097

[43] Chen SH, Hung WC, Wang P, Paul C, Konstantopoulos K. Mesothelin binding to CA125/MUC16 promotes pancreatic cancer cell motility and invasion via MMP-7 activation. Scientific reports. 2013;3:1870. Epub 2013/05/23. doi: 10.1038/srep01870 . 23694968 PMC3660778

[44] Shukla SK, Gunda V, Abrego J, Haridas D, Mishra A, Souchek J, et al. MUC16-mediated activation of mTOR and c-Myc reprograms pancreatic cancer metabolism. Oncotarget. 2015;6(22):19118–31. doi: 10.18632/oncotarget.4078 . 26046375 PMC4662479

[45] Mehla K, Singh PK. MUC1: a novel metabolic master regulator. Biochimica et biophysica acta. 2014;1845(2):126–35. Epub 2014/01/11. doi: 10.1016/j.bbcan.2014.01.001 . 24418575 PMC4045475

[46] Singh PK, Behrens ME, Eggers JP, Cerny RL, Bailey JM, Shanmugam K, et al. Phosphorylation of MUC1 by Met modulates interaction with p53 and MMP1 expression. The Journal of biological chemistry. 2008;283(40):26985–95. Epub 2008/07/14. doi: 10.1074/jbc.M805036200 . 18625714 PMC2556014

[47] Chaika NV, Gebregiworgis T, Lewallen ME, Purohit V, Radhakrishnan P, Liu X, et al. MUC1 mucin stabilizes and activates hypoxia-inducible factor 1 alpha to regulate metabolism in pancreatic cancer. Proceedings of the National Academy of Sciences of the United States of America. 2012;109(34):13787–92. Epub 2012/08/06. doi: 10.1073/pnas.1203339109 . 22869720 PMC3427054

[48] Shukla SK, Purohit V, Mehla K, Gunda V, Chaika NV, Vernucci E, et al. MUC1 and HIF-1alpha Signaling Crosstalk Induces Anabolic Glucose Metabolism to Impart Gemcitabine Resistance to Pancreatic Cancer. Cancer cell. 2017;32(1):71–87.e7. doi: 10.1016/j.ccell.2017.06.004 . 28697344 PMC5533091

[49] Gunda V, Souchek J, Abrego J, Shukla SK, Goode GD, Vernucci E, et al. MUC1-Mediated Metabolic Alterations Regulate Response to Radiotherapy in Pancreatic Cancer. Clinical cancer research: an official journal of the American Association for Cancer Research. 2017;23(19):5881–91. Epub 2017/07/18. doi: 10.1158/1078-0432.CCR-17-1151 . 28720669 PMC5626603

[50] Suh H, Pillai K, Morris DL. Mucins in pancreatic cancer: biological role, implications in carcinogenesis and applications in diagnosis and therapy. American journal of cancer research. 2017;7(6):1372–83. . 28670497 PMC5489784

[51] Ganguly K, Bhatia R, Rauth S, Kisling A, Atri P, Thompson C, et al. Mucin 5AC Serves as the Nexus for β-Catenin/c-Myc Interplay to Promote Glutamine Dependency During Pancreatic Cancer Chemoresistance. Gastroenterology. 2022;162(1):253–68.e13. doi: doi: 10.1053/j.gastro.2021.09.017 34534538 PMC8678212

